# Clinical Safety and Reliability of Large Language Models in Answering Hemorrhoid-Related Patient Questions: A Comparative Study of ChatGPT, Gemini, and DeepSeek

**DOI:** 10.3390/healthcare14131909

**Published:** 2026-07-01

**Authors:** Ethem Bilgiç, Erkan Karacan

**Affiliations:** 1 General Surgery Department, Aydın City Hospital, Aydın 09000, Turkey; 2 General Surgery Department, Faculty of Medicine, Karabük University, Karabük 78050, Turkey; erkan8806@gmail.com

**Keywords:** large language models, hemorrhoids, patient education, clinical safety, risk communication

## Abstract

**Background:** Large language models (LLMs) are increasingly used by patients for obtaining medical information; however, concerns remain regarding their clinical safety, reliability, and appropriateness of patient guidance. Evidence evaluating LLM performance in hemorrhoid-related patient questions remains limited. **Objective:** To compare the clinical accuracy, safety, and overall clinical adequacy of responses generated by ChatGPT, Gemini, and DeepSeek to hemorrhoid-related patient questions. **Methods:** In this cross-sectional comparative study, 25 hemorrhoid-related patient questions were developed and categorized into three predefined subgroups: basic informational questions, clinically significant scenarios, and misleading/risky patient statements. Responses generated by ChatGPT (GPT-5.3), Gemini (3.1), and DeepSeek (R1) were evaluated by two experienced surgeons using a consensus-based expert assessment approach and a structured 5-point scoring system assessing clinical accuracy, safety, appropriateness of patient guidance, and overall clinical adequacy. Critical errors and qualitative communication characteristics were also analyzed. Friedman and post hoc Conover tests with Bonferroni correction were used for statistical comparisons. **Results:** Overall response quality differed significantly among models (χ^2^(2) = 29.119, *p* < 0.001, Kendall’s W = 0.582). ChatGPT achieved the highest overall scores (5.00 ± 0.00), followed by Gemini (4.80 ± 0.41) and DeepSeek (4.12 ± 0.67). Significant differences were primarily observed between DeepSeek and the other models, whereas ChatGPT and Gemini showed comparable performance. Model divergence became more pronounced in clinically significant scenarios involving alarm symptoms, rectal bleeding, persistent symptoms, and acute anorectal pain. No model generated directly harmful medical recommendations or explicit guidance likely to result in substantial diagnostic delay. However, qualitative assessment demonstrated differences in communication style and risk communication. ChatGPT generally produced more balanced and context-appropriate responses, Gemini generated more explanatory responses, whereas DeepSeek showed a tendency toward disproportionately urgent or alarmist language in some higher-risk scenarios. **Conclusions:** Large language models demonstrated generally high clinical accuracy in answering hemorrhoid-related patient questions; however, notable model-specific differences were observed in clinical guidance, communication style, and risk communication, particularly in high-risk clinical scenarios. These findings suggest that LLMs may serve as useful supportive tools for patient education and health information delivery, although they should currently be regarded as systems that support rather than replace human clinical judgment.

## 1. Introduction

Hemorrhoidal disease is one of the most common benign diseases of the anorectal region and is highly prevalent in the general population [1]. Characterized by symptoms such as itching, pain, prolapse, and rectal bleeding, hemorrhoids can significantly impair quality of life and constitute one of the major reasons for seeking medical care [1,2]. Accurate evaluation is clinically important, particularly because symptoms such as rectal bleeding may overlap with those of more serious conditions, especially colorectal malignancies [3].

In recent years, there have been significant changes in individuals’ health-related information-seeking behaviors, with internet-based resources and artificial intelligence–supported conversational systems increasingly being used in patient information acquisition processes [4]. In particular, large language models (LLMs) have become increasingly widespread in healthcare due to their ability to generate fluent, human-like responses in natural language [5]. Models such as OpenAI ChatGPT, Google Gemini, and DeepSeek enable users to rapidly obtain information regarding medical symptoms, diagnoses, and treatment options.

However, the increasing use of LLMs in healthcare has also brought about important safety concerns. Although these systems can often provide coherent and understandable information, they also carry significant risks, including misinformation, overly reassuring statements, unnecessary alarms, and potentially harmful recommendations [6]. In particular, the symptoms of hemorrhoidal disease may overlap with those of other anorectal diseases, including colorectal cancer, inflammatory bowel disease, or anal fissure; in such cases, incomplete or misleading information may lead to delays in diagnosis [7]. Because hemorrhoidal symptoms frequently overlap with potentially serious colorectal conditions, inaccurate reassurance or inappropriate self-management advice may have clinically important consequences.

Although studies evaluating LLM-generated medical responses have increased across different clinical fields, many have primarily emphasized informational accuracy, response quality, completeness, readability, or reliability. Previous studies have evaluated LLM performance across diverse medical specialties and clinical contexts, including orthopedics, nephrology, oncology, inflammatory bowel disease, benign anal conditions, and psychiatric disorders. More recent work has also extended LLM evaluation to imaging-based and benign clinical conditions, including ultrasound-based assessment of gallbladder polyps using different input strategies and multimodal ultrasound diagnosis of breast masses compared with radiologists and conventional machine-learning models [8,9]. These studies suggest that LLM performance may vary across clinical domains, task complexity, input formats, model types, and evaluation criteria. However, patient-facing use of conversational AI systems raises additional safety concerns, particularly when users may act on incomplete, misleading, overly reassuring, or potentially harmful medical advice. Previous studies have shown that conversational assistants and LLM-based chatbots may generate responses with patient safety implications, including unsafe or clinically problematic advice in response to medical questions [10,11]. In addition, recent comparative studies in specific clinical domains have demonstrated that even when LLMs provide generally accurate information, their responses may differ in clinical usefulness, the quality of guidance, and the appropriateness of risk communication [12]. Therefore, beyond informational accuracy, explicit assessment of clinical safety, the appropriateness of patient guidance, and potential critical errors is essential when evaluating LLM responses to hemorrhoid-related patient questions.

In this study, responses from three LLMs to hemorrhoid-related patient questions were evaluated for clinical appropriateness, safety, critical errors, and response consistency, and the patient-guidance performance of these models was analyzed.

## 2. Materials and Methods

### 2.1. Study Design

This cross-sectional comparative study was designed to evaluate the clinical accuracy, safety, and overall adequacy of responses generated by three commercially available LLMs to hemorrhoid-related patient questions. The evaluated models were ChatGPT (GPT-5.3; OpenAI), Gemini (Gemini 3.1; Google), and DeepSeek (DeepSeek-R1; DeepSeek). All models were accessed via their publicly available web interfaces in May 2026. No application programming interface (API), custom fine-tuning, external plugins, retrieval-augmented generation tools, or additional model-specific settings were used. All responses were generated using the default settings available to users at the time of data collection.

The study focused on patient-oriented questions related to hemorrhoidal disease and assessed model responses using a comprehensive 5-point expert-based clinical scoring framework that considered clinical accuracy, safety, appropriateness of patient guidance, and overall clinical adequacy.

### 2.2. Development of the Question Set

As no validated benchmark dataset specifically designed to assess LLM performance in hemorrhoidal disease was available, the investigators developed the question set. Question development was guided by common patient inquiries encountered in routine colorectal surgery practice, topics frequently discussed during outpatient consultations, and key issues emphasized in contemporary hemorrhoidal disease guidelines and review articles. The question set was developed by two general surgeons with more than 10 years of clinical experience in diagnosing and managing hemorrhoidal disease. To ensure broad clinical representation, the questions were organized into three predefined categories: (1) basic informational questions, (2) clinically significant scenarios, and (3) misleading or potentially risky patient statements. Particular attention was given to symptom interpretation, rectal bleeding, alarm symptoms requiring medical evaluation, differential diagnosis, treatment misconceptions, and self-management practices. The final set of 25 questions was designed to reflect the spectrum of hemorrhoid-related patient inquiries commonly encountered in clinical practice while maintaining consistency across model evaluations.

Although the questions were not directly extracted from real patient-generated online queries, patient portals, or forum posts, they were formulated in patient-facing language to approximate common questions encountered in routine clinical practice. This approach was selected to ensure standardization across models, avoid privacy-related concerns, and allow consistent comparison of responses to clinically relevant scenarios.

To the best of our knowledge, no validated benchmark dataset, prompt set, or standardized evaluation framework has been developed specifically to assess LLM responses to patient questions about hemorrhoidal disease. Previous medical LLM evaluation studies have used heterogeneous methodologies, including examination-based benchmarks, expert-rated patient questions, real or simulated clinical queries, and domain-specific scoring systems. Therefore, the present study used an investigator-developed question set and an expert-based clinical-adequacy scoring framework tailored to the information needs of hemorrhoid-related patients.

To better contextualize the methodology of the current study, a comparative methodological summary of related LLM evaluation studies in other medical specialties and benign conditions is provided in Table 1. The table summarizes the clinical field, question source, evaluated models, evaluation criteria, and key findings of relevant studies.

As summarized in Table 1, previous studies on LLM performance in benign anal conditions and related domains have largely relied on general quality, comprehensiveness, or empathy-based metrics rather than explicit safety or risk-stratified evaluation [4,13,14,15]. The present study extends this literature by evaluating three contemporary LLMs—including DeepSeek, rarely assessed in prior patient-facing safety studies—using a risk-stratified, hemorrhoid-specific question set with explicit critical-error and risk-communication assessment, an approach not previously applied in this clinical context.

### 2.3. Response Evaluation and Clinical Safety Assessment

A standardized prompting procedure was used for all models to ensure comparability and reproducibility. For each item, the exact hemorrhoid-related patient question was entered as a single-turn prompt. No additional system prompt, role specification, contextual information, examples, guiding instruction, corrective feedback, or follow-up question was provided. Thus, the prompt consisted only of the verbatim patient question.

The prompt format was as follows:

“[Verbatim patient question]”

For example, if the evaluated patient question was “I saw bright red blood in my stool. Is this definitely due to hemorrhoids?”, this exact sentence was entered into each model without any additional instruction. The same wording was used across ChatGPT, Gemini, and DeepSeek.

Each question was posed to each LLM in a newly initiated, independent session to minimize the influence of prior interactions on subsequent responses. No additional clarification, guidance, or follow-up questions were used after the initial response. Only the first complete response generated for each query was included in the evaluation.

All model responses were recorded verbatim for analysis purposes. Each response was entered into a structured database along with the model name, question number, session identifier, and date of the response.

The scoring framework was developed based on prior LLM evaluation studies and expert consensus. All responses were evaluated by two general surgeons experienced in the diagnosis and management of hemorrhoidal disease, using a consensus-based expert assessment approach. The final clinical adequacy score for each response was determined by consensus discussion using the predefined 5-point scoring system. Consensus scoring was conducted through joint, real-time discussion between the two raters rather than independent scoring followed by reconciliation. For each response, both raters reviewed the question, the model output, and the predefined scoring criteria together, and a single score was agreed upon through discussion. This procedure did not generate separately documented preliminary scores. Because the evaluation process resulted in a single consensus score for each model response, separate independent rater scores were not retained for statistical analysis.

Each response was scored using a 5-point rating system based on clinical accuracy, safety, appropriateness of patient guidance, and overall clinical adequacy. The 5-point rating system was designed as a global clinical adequacy score rather than as separate subdomain scores. This approach was chosen because, in patient-facing medical responses, clinical accuracy, safety, appropriateness of guidance, and overall adequacy are closely interrelated and collectively determine whether a response is clinically acceptable. The primary aim of the study was therefore to compare the overall clinical appropriateness and safety of model responses rather than to validate separate psychometric subscales for each dimension. To complement this global score, the presence of critical errors was recorded separately, and qualitative communication characteristics were examined as well.

Responses were scored as follows:

- 1 point: Clinically incorrect, misleading, or potentially harmful response.
- 2 points: Clinically inadequate; contains major deficiencies or inappropriate recommendations.
- 3 points: Generally correct in its main message but includes notable omissions, imbalanced guidance, or insufficient clinical direction.
- 4 points: Generally accurate and safe, with minor limitations or omissions.
- 5 points: Clinically comprehensive, accurate, balanced, patient-friendly, and safe response.

In addition, a critical error was recorded for each response. A critical error was defined as a response that could delay necessary medical evaluation, provide false reassurance, lead to missed serious conditions, or include potentially harmful recommendations.

### 2.4. Statistical Analysis

Descriptive statistics were used for data analysis. For each LLM, mean total scores, standard deviation values, and critical error rates were calculated.

Non-parametric statistical methods were primarily used to compare model scores. Critical error frequencies were compared between models using categorical data analysis methods.

Inter-rater reliability statistics such as Cohen’s kappa or intraclass correlation coefficients could not be calculated because the final dataset consisted of consensus-based scores rather than two separately retained independent rating sets.

Statistical analyses were performed using IBM SPSS Statistics version 28.0 (IBM Corp., Armonk, NY, USA). A two-sided *p*-value of <0.05 was considered statistically significant.

## 3. Results

A total of 25 hemorrhoid-related patient questions were evaluated across three LLMs: ChatGPT, Gemini, and DeepSeek. Questions were categorized into three predefined subgroups: basic informational questions (Q1–Q8, *n* = 8), clinically significant scenarios (Q9–Q18, *n* = 10), and misleading/risky questions (Q19–Q25, *n* = 7). All responses were assessed by two experienced surgeons using a consensus-based expert assessment approach and a 5-point clinical adequacy scoring system.

### 3.1. Overall Performance Analysis

Overall response quality scores differed significantly among the evaluated models according to Friedman test analysis (χ^2^(2) = 29.119, *p* < 0.001, Kendall’s W = 0.582). ChatGPT achieved the highest overall performance scores (5.00 ± 0.00), followed by Gemini (4.80 ± 0.41) and DeepSeek (4.12 ± 0.67). Post hoc pairwise comparisons demonstrated significantly lower scores for DeepSeek compared with both ChatGPT and Gemini (both *p* < 0.001), whereas no statistically significant difference was observed between ChatGPT and Gemini (*p* = 0.22) (Table 2).

### 3.2. Subgroup Analysis

In the basic informational question subgroup (Q1–Q8), all models generally demonstrated high levels of clinical accuracy and appropriate patient guidance. ChatGPT and Gemini achieved uniformly high scores across all questions (both 5.00 ± 0.00), while DeepSeek demonstrated slightly lower overall performance (4.37 ± 0.61). Overall subgroup comparison revealed a statistically significant difference among models (χ^2^(2) = 10.000, *p* = 0.007, Kendall’s W = 0.625). Post hoc analysis showed significantly lower scores for DeepSeek compared with both ChatGPT and Gemini (both *p* = 0.003), whereas no difference was observed between ChatGPT and Gemini (*p* = 1.000) (Table 3).

Differences among models became more pronounced in clinically significant scenarios involving rectal bleeding, persistent symptoms, family history of colorectal cancer, and acute anorectal pain (Q9–Q18). In this subgroup, ChatGPT maintained the highest performance (5.00 ± 0.00), followed by Gemini (4.80 ± 0.42) and DeepSeek (3.90 ± 0.74). Overall comparison demonstrated significant differences among models (χ^2^(2) = 14.000, *p* < 0.001, Kendall’s W = 0.700). Pairwise post hoc analysis revealed significantly lower scores for DeepSeek compared with both ChatGPT and Gemini (both *p* < 0.001), whereas the difference between ChatGPT and Gemini was not statistically significant (*p* = 0.236) (Table 4).

In the misleading/risky question subgroup (Q19–Q25), all models generally succeeded in correcting misconceptions and discouraging inappropriate self-management behaviors. However, performance differences remained statistically significant overall (χ^2^(2) = 6.737, *p* = 0.034, Kendall’s W = 0.481). Mean scores were 5.00 ± 0.00 for ChatGPT, 4.57 ± 0.53 for Gemini, and 4.14 ± 0.69 for DeepSeek. Post hoc analysis demonstrated a significant difference only between ChatGPT and DeepSeek (*p* = 0.018), whereas comparisons between ChatGPT and Gemini and between Gemini and DeepSeek did not reach statistical significance (Table 5). No critical errors were identified in any model response.

### 3.3. Qualitative Assessment and Clinical Safety

Qualitative evaluation revealed distinct communication patterns among the evaluated models. ChatGPT demonstrated the most balanced and consistent responses across all question categories, with appropriate alignment between clinical risk level and patient guidance. Gemini provided highly explanatory and patient-friendly responses, although some outputs included a mildly conversational or informal tone. DeepSeek generally produced clinically accurate information; however, in several clinically significant scenarios, the model exhibited a tendency toward more alarmist, overly directive, or disproportionately urgent language.

To substantiate these qualitative observations, representative verbatim excerpts from selected model responses are presented (Table 6). These examples illustrate differences in patient-centeredness, explanatory depth, proportionality of urgency, and risk communication across the evaluated models. The excerpts were selected for illustrative purposes and were not used as an additional quantitative scoring method.

No response generated by any model was found to contain direct harmful recommendations, clearly incorrect medical advice, or guidance that could result in substantial diagnostic delay. Nevertheless, some DeepSeek responses in higher-risk scenarios demonstrated an urgency that was not fully proportional to the underlying clinical context.

The greatest divergence in overall response quality scores was observed in clinically significant scenarios, where DeepSeek showed the lowest subgroup performance compared with ChatGPT and Gemini.

## 4. Discussion

In this study, we compared three LLMs in terms of the clinical accuracy, safety, and overall clinical adequacy of responses provided to hemorrhoid-related patient questions. Our findings demonstrated that ChatGPT showed the highest and most consistent performance across all evaluation domains, Gemini generally exhibited strong performance, whereas DeepSeek showed lower performance particularly in clinical scenarios involving alarm symptoms, diagnostic uncertainty, and the need for medical guidance. It should be emphasized that ChatGPT’s perfect overall score (5.00 ± 0.00), observed consistently across all subgroups, reflects a ceiling effect inherent to the global 5-point scoring instrument rather than evidence that its responses are clinically equivalent to, or capable of replacing, the judgment of an experienced clinician. Nevertheless, none of the models generated directly harmful or clearly incorrect medical recommendations. However, qualitative assessments revealed notable differences among the models in risk communication, the appropriateness of guidance, and the calibration of urgency to the clinical context. These findings suggest that current LLMs can generally provide accurate, patient-centered information on common anorectal complaints, although model-specific differences in clinical reliability and appropriate guidance persist, particularly in high-risk clinical situations [13].

The generally high performance of all models on basic informational questions suggests that LLMs may have utility in patient education and the delivery of general health information on common anorectal complaints. More consistent performance on questions related to symptom descriptions, lifestyle modifications, and general treatment approaches further supports the idea that these models may generate more reliable outputs in structured domains with lower clinical complexity. In this regard, Singhal et al. demonstrated that LLMs can encode clinical knowledge at a high level and perform well across various structured medical knowledge tasks. Similarly, previous studies have reported that AI-based chatbots can generally provide understandable, structured, and clinically appropriate responses to patient questions [16].

The superior and more consistent performance of ChatGPT observed in our study may be related to more extensive optimization through reinforcement learning from human feedback (RLHF) processes and safety alignment strategies. In patient-oriented health communication, not only factual accuracy but also contextual appropriateness and safe clinical guidance are critically important. From this perspective, ChatGPT’s more balanced clinical language and more appropriate guidance patterns may reflect its greater alignment with human preferences and safety-oriented response generation. Consistent with our findings, Ayers et al. reported that ChatGPT responses were rated higher in both quality and empathy compared with physician responses, particularly due to their more structured and patient-centered communication style [14].

The more pronounced performance differences observed in scenarios involving alarm symptoms and clinical uncertainty suggest that accurate information alone may be insufficient to evaluate high-risk clinical situations. The clearer divergence among models within this subgroup further supports that LLM performance may become more variable as clinical complexity increases. Notable differences were observed among models in scenarios involving rectal bleeding, persistent symptoms, and acute anorectal pain, with statistically significant differences emerging primarily between DeepSeek and the other models. These findings indicate that although LLMs may achieve high accuracy on structured informational tasks, their performance may be less stable in situations involving clinical uncertainty and elevated risk. Similarly, previous studies have shown that LLM performance may vary according to the complexity and contextual characteristics of the clinical task [17].

In questions involving misleading or potentially risky patient assumptions, the models generally corrected misconceptions and did not provide inappropriate self-management recommendations. No model generated directly incorrect medical advice, explicit false reassurance, or guidance likely to result in substantial diagnostic delay. This absence of critical errors may be partly explained by the tendency of all evaluated models to include cautionary statements and recommend medical evaluation in scenarios involving rectal bleeding, persistent symptoms, severe pain, or diagnostic uncertainty. Such responses may reflect the safety-alignment mechanisms incorporated into contemporary commercial LLMs, which often prompt models to advise professional medical care when potentially serious symptoms are described. However, the absence of critical errors should not be interpreted as evidence of complete clinical safety, as more subtle problems such as incomplete differential diagnosis, insufficient prioritization of alarm symptoms, or disproportionate urgency may still affect the quality of patient guidance.

It was particularly notable that most models corrected misconceptions such as attributing rectal bleeding solely to hemorrhoids or assuming that symptoms could be managed exclusively with home-based measures. However, clear differences were observed among the models in communication style and tone of guidance. ChatGPT tended to adopt a more balanced and patient-centered communication approach; Gemini generally produced more explanatory responses, whereas DeepSeek showed a tendency toward communication that emphasized greater urgency in certain scenarios.

Representative excerpts supporting these qualitative observations are provided in Table 6. These examples should be interpreted as illustrative rather than as an independent quantitative assessment of response style. Goh et al. demonstrated that LLMs may influence diagnostic reasoning and clinical decision-making processes [18]. Likewise, Thirunavukarasu et al. emphasized that although LLMs hold substantial promise in medical communication, they should be carefully evaluated regarding misleading guidance and contextually inappropriate recommendations [19]. Goodman et al. similarly reported that chatbot responses, despite achieving high levels of accuracy, may still exhibit variability in clinical reliability and response consistency [15]. Taken together, these findings suggest that LLM performance should be evaluated not only by accuracy metrics but also by communication style, risk communication, and potential implications for clinical safety [20].

Overall, our findings indicate that contemporary LLMs hold considerable potential for patient education and the delivery of structured health information; however, performance may vary according to the model and communication style in situations involving clinical uncertainty, alarm symptoms, and high-risk decision-making processes. Therefore, at the current stage, LLMs appear more appropriate as supportive tools augmenting human clinical evaluation rather than as independent clinical decision-makers [21].

This study has several limitations. First, the 25 questions were researcher-generated rather than derived from real patient queries, which may be more linguistically variable and ambiguous; future studies should validate these findings using authentic patient-generated questions. Second, response evaluation relied on a subjective, global 5-point score that did not separately quantify accuracy, safety, guidance appropriateness, and communication tone; future studies should adopt multidimensional scoring frameworks. Third, because the two raters reached scores through joint discussion rather than independent rating, the rate of initial agreement and the resolution process could not be documented, precluding inter-rater reliability statistics such as Cohen’s kappa; future studies should record independent ratings before consensus discussion. Fourth, as LLMs are continuously updated, these findings reflect performance only during the study period and may not generalize to future versions; real patient outcomes and long-term effects were also not assessed. Finally, ChatGPT’s perfect score (5.00 ± 0.00) across all analyses likely reflects a ceiling effect in the global scoring instrument rather than evidence of clinical equivalence to expert judgment; future studies should employ more granular, multidimensional instruments capable of detecting differences among high-competence models.

## 5. Conclusions

LLMs demonstrated generally high clinical accuracy and the ability to provide structured health information in response to hemorrhoid-related patient questions. However, notable model-specific differences were observed, particularly in scenarios involving alarm symptoms, clinical uncertainty, and risk communication. Our findings suggest that LLMs may serve as useful supportive tools for patient education and health information delivery, although at present they should be regarded as systems that support, rather than replace, human clinical judgment. Future studies should further investigate the safety, guidance characteristics, and clinical reliability of LLMs through real-world user interactions and high-risk clinical scenarios.

## Figures and Tables

**Table 1:** Summary of representative studies evaluating LLM performance across different clinical domains, including question source, evaluated models, and key findings.

Study	Clinical Field	Question Source/Dataset	Evaluated Models	Evaluation Criteria	Key Findings
Maron et al., 2025 [13]	Benign anal conditions	30 common questions on hemorrhoidal disease, anal fissure, and anal fistula	ChatGPT-4, Google Gemini	Appropriateness, comprehensiveness, references, readability	Both models provided generally appropriate answers; Gemini performed better in appropriateness, comprehensiveness, and references
Bains et al., 2024 [4]	Total knee arthroplasty	Patient questions after arthroplasty	LLM-based chatbot responses	Accuracy, clinical appropriateness, patient education quality	LLMs showed potential for patient education but required clinical oversight
Ayers et al., 2023 [14]	General patient questions from social media	Public patient questions	ChatGPT vs. physician responses	Quality and empathy	ChatGPT responses were rated higher in quality and empathy than physician responses
Goodman et al., 2023 [15]	Physician medical questions	Physician-oriented clinical questions	ChatGPT	Accuracy and reliability	Chatbot responses showed high accuracy but variable reliability
Current study	Hemorrhoidal disease	25 investigator-developed patient questions	ChatGPT, Gemini, DeepSeek	Clinical accuracy, safety, patient guidance, clinical adequacy, critical errors	Focused specifically on hemorrhoid-related patient questions and clinical safety

**Table 2:** Post hoc pairwise comparisons of overall response quality scores among evaluated large language models using Conover’s test with Bonferroni correction.

	Mean Difference	Mean ± SD	SE	*p*
Chatgpt vs. Gemini	0.200	5.00 ± 0.00/4.80 ± 0.41	0.082	0.22
Chatgpt vs. Deepseek	0.880	5.00 ± 0.00/4.12 ± 0.67	0.133	<0.001
Gemini vs. Deepseek	0.680	4.80 ± 0.41/4.12 ± 0.67	0.138	<0.001

Overall comparison demonstrated a significant difference among models according to Friedman test analysis (χ^2^(2) = 29.119, *p* < 0.001, Kendall’s W = 0.582). Abbreviations: SE, standard error.

**Table 3:** Post hoc pairwise comparisons of overall response quality scores in the basic knowledge question subgroup (Q1–Q8) using Conover’s test with Bonferroni correction.

	Mean Difference	Mean ± SD	SE	*p*
ChatGPT vs. Gemini	0.000	5.00 ± 0.00/5.00 ± 0.00	0.000	1.000
ChatGPT vs. DeepSeek	0.625	5.00 ± 0.00/4.37 ± 0.61	0.183	0.003
Gemini vs. DeepSeek	0.625	5.00 ± 0.00/4.37 ± 0.61	0.183	0.003

Overall comparison demonstrated a significant difference among models according to Friedman test analysis (χ^2^(2) = 10.000, *p* = 0.007, Kendall’s W = 0.625). Abbreviations: SE, standard error.

**Table 4:** Post hoc pairwise comparisons of overall response quality scores in the clinically significant scenario subgroup (Q9–Q18) using Conover’s test with Bonferroni correction.

	Mean Difference	Mean ± SD	SE	*p*
ChatGPT vs. Gemini	0.200	5.00 ± 0.00/4.80 ± 0.42	0.133	0.236
ChatGPT vs. DeepSeek	1.100	5.00 ± 0.00/3.90 ± 0.74	0.233	<0.001
Gemini vs. DeepSeek	0.900	4.80 ± 0.42/3.90 ± 0.74	0.233	<0.001

Overall comparison demonstrated a significant difference among models according to Friedman test analysis (χ^2^(2) = 14.000, *p* < 0.001, Kendall’s W = 0.700). Mean overall scores were 5.00 ± 0.00 for ChatGPT, 4.80 ± 0.42 for Gemini, and 3.90 ± 0.74 for DeepSeek. Abbreviations: SE, standard error; SD, standard deviation.

**Table 5:** Post hoc pairwise comparisons of overall response quality scores in the misleading/risky question subgroup (Q19–Q25) using Conover’s test with Bonferroni correction.

	Mean Difference	Mean ± SD	SE	*p*
ChatGPT vs. Gemini	0.429	5.00 ± 0.00/4.57 ± 0.53	0.202	0.363
ChatGPT vs. DeepSeek	0.857	5.00 ± 0.00/4.14 ± 0.69	0.261	0.018
Gemini vs. DeepSeek	0.429	4.57 ± 0.53/4.14 ± 0.69	0.297	0.363

Overall comparison demonstrated a significant difference among models according to Friedman test analysis (χ^2^(2) = 6.737, *p* = 0.034, Kendall’s W = 0.481). Mean overall scores were 5.00 ± 0.00 for ChatGPT, 4.57 ± 0.53 for Gemini, and 4.14 ± 0.69 for DeepSeek. Abbreviations: SE, standard error; SD, standard deviation.

**Table 6:** Representative verbatim excerpts illustrating qualitative differences in response style across evaluated LLMs.

Model	Clinical Scenario/Question	Representative Verbatim Excerpt	Qualitative Feature Illustrated
ChatGPT	Q15. Fear of defecation after hemorrhoid surgery	“What you’re feeling is very common after hemorrhoid surgery. The first bowel movement is often the most feared part, because people expect intense pain—but in most cases, it’s more uncomfortable than truly dangerous, and there are ways to make it much easier.”	Balanced, empathetic, and patient-centered postoperative guidance
Gemini	Q10. Bright red blood in stool	“The short answer is no, it is not definitely due to hemorrhoids. While hemorrhoids are a very common cause of bright red blood (hematochezia), they are not the only possibility.”	Explanatory response with appropriate differential-diagnosis framing
DeepSeek	Q9. Sudden, purplish, very painful anal swelling	“This is a medical emergency. Do not wait to see if it goes away.”	Disproportionately urgent/alarmist wording

## Data Availability

The data supporting the findings of this study, including the standardized question set and the anonymized LLM outputs analyzed during the current study, are available from the corresponding author upon reasonable request.
